# Migrasome regulator TSPAN4 shapes the suppressive tumor immune microenvironment in pan-cancer

**DOI:** 10.3389/fimmu.2024.1419420

**Published:** 2024-12-11

**Authors:** Lin-jian Wang, Ruiyan Xu, Yangyang Wu

**Affiliations:** ^1^ Trauma Research Center, Zhengzhou Central Hospital Affiliated to Zhengzhou University, Zhengzhou, China; ^2^ Department of Neurosurgery, Zhengzhou Central Hospital Affiliated to Zhengzhou University, Zhengzhou, China; ^3^ Zhengzhou Central Hospital Affiliated to Zhengzhou University, Zhengzhou, China

**Keywords:** TSPAN4, pan-cancer, prognosis, glioma, tumor immune microenvironment

## Abstract

**Background:**

Migrasomes are newly identified organelles on the retracting fibers of migrating cells, involved in releasing signaling molecules, expelling damaged mitochondria, and facilitating intercellular communication through phagocytosis. TSPAN4, a key regulator of migrasome formation, is a valuable marker for visualizing these organelles. However, its role in cancer remains unclear.

**Methods:**

We analyzed TSPAN4 expression and its prognostic significance across multiple cancers using TCGA Pan-Cancer (PANCAN), and TCGA TARGET GTEx datasets. The relationship between TSPAN4 and tumor heterogeneity, stemness, and the immunosuppressive tumor microenvironment was explored through RNA-seq and scRNA-seq data. In addition, we examined TSPAN4's role in glioma, focusing on migrasome formation, cell proliferation, and macrophage polarization.

**Results:**

Our analysis reveals that TSPAN4 is aberrantly expressed in various tumors, likely linked to its methylation status. It correlates with tumor heterogeneity, stemness, and a suppressive immune microenvironment. In glioma, TSPAN4 enhances cell proliferation and promotes macrophage polarization toward the immunosuppressive M2 phenotype.

**Conclusions:**

TSPAN4, as a migrasome regulator, plays a crucial role in shaping the immunosuppressive tumor microenvironment in pan-cancer.

## Introduction

Recently, a new cellular organelle with a pomegranate-like morphology was discovered on the retraction fibers of migrating cells and named the migrasome due to its formation’s complete dependence on cell migration (1). The migrasome, akin to a cargo space, contains a diverse array of signaling molecules, including chemokines, cytokines, and growth factors, which are released into the environment upon migrasome rupture (1). In addition, the migrasome can be taken up by surrounding recipient cells, facilitating the transfer of its mRNA and proteins between donor and recipient cells (2). This confers upon migrasomes the capability to integrate spatial and biological information, thereby regulating processes such as organ morphogenesis and angiogenesis (3, 4).

Tetraspanin 4 (TSPAN4) is a member of the transmembrane 4 superfamily (also known as the tetraspanin family). The majority of these members are cell surface proteins comprising four hydrophobic structural domains. They are involved in a variety of cellular processes, including adhesion, migration, membrane-remodeling, and signal transduction, and play key roles in the pathogenesis of diseases such as the immune system, the nervous system, cancer, and infection (5–7). TSPAN4 has been shown to be a valuable marker for visualizing migrasomes in migrating cells, and its overexpression markedly enhances migrasome formation (8). Prior research indicated that TSPAN4 drives GBM progression by regulating EGFR stability (9). In addition, migrasomes possess the capacity to alleviate cellular stress in glioblastoma (GBM) (10). However, research into the role of TSPAN4 in cancer remains largely uncharted territory and awaits further exploration.

In this study, we comprehensively analyzed the expression and function of TSPAN4 in pan-cancer. TSPAN4 exhibited dysregulated expression in 31 tumor tissues compared with their corresponding normal tissues, with alterations changes in expression were linked to DNA methylation. Elevated expression of TSPAN4 resulted in diminished overall survival in GBM, LGG, GBMLGG, ACC, STAD, LUSC, and BLCA. Furthermore, we observed a robust correlation between TSPAN4 and genomic heterogeneity, stemness, and the tumor microenvironment in TSPAN4-sensitive tumors, particularly in GBMLGG and LUSC. TSPAN4 expression was positively correlated with immunomodulators including PD-L1, CTLA-4, and LAG-3, as well as the infiltration of immunosuppressive cells such as Macrophages M2, exhausted T-cells and T cells regulatory (Tregs), implying its involvement in shaping a suppressive tumor immune microenvironment. Furthermore, we demonstrated that TSPAN4 promoted the proliferation of glioma cells and induced the polarization of macrophages toward the immunosuppressive M2 type.

## Methods

### Samples and datasets

The merged dataset TCGA GBMLGG, the unified normalized pan-cancer dataset TCGA Pan-Cancer (PANCAN) and TCGA TARGET GTEx were downloaded from the University of California Santa Cruz (UCSC) Xena (https://xenabrowser.net/) (11). The mRNAseq_325 and mRNAseq_693 glioma datasets were downloaded from the Chinese Glioma Genome Atlas (CGGA) data portal (http://www.cgga.org.cn/) (12). The promoter methylation data were obtained from The University of ALabama at Birmingham CANcer data analysis Portal (UALCAN, http://ualcan.path.uab.edu/) (13, 14). Single-cell RNA-seq (scRNA-seq) data (GSE141460) were retrieved from the Gene Expression Omnibus (GEO) (15). Cell type annotations, analyses of differential gene expression, and cell-cell interactions were conducted using Tumor Immune Single-cell Hub 2 (TISCH2) and cell type annotations at the single cell level as well as analyses of differential gene expression, and cell-cell interactions were performed on Tumor Immune Single-cell Hub 2 (TISCH2) (16).

### Survival analysis of TSPAN4 in pan-cancer

A Cox proportional hazards regression model was constructed using the “coxph” function of the R package survival to examine the relationship between gene expression and prognosis in each tumor. For Kaplan-Meier analysis, we calculated the optimal cutoff value for TSPAN4 using the R package maxstat (Maximally selected rank statistics with several p-value approximations version:0.7-25). Patients were subsequently divided into two groups based on this optimal cutoff value, and the prognostic differences between these groups were further analyzed using the “survfit” function from the R package survival.

### Analysis of tumor heterogeneity and stemness

The R software package “maftools” was used to calculate the Mutant-allele tumor heterogeneity (MATH) and Tumor mutation burden (TMB) for each sample. Microsatellite instability (MSI) data were derived from the **Supplementary Files** of Bonneville’s study (17). Loss of heterozygosity (LOH), ploidy, Neoantigen (Neo), Homologous recombination deficiency (HRD) and purity were extracted from the Thorsson’s study (18). According to previous study, eight tumor stemness scores were calculated, encompassing DNAss (DNA methylation), EREG-METHs (Epigenetically regulated DNA methylation), DMPss (Differentially methylated probes), ENHs (Enhancer Elements/DNA methylation), RNAss (based on RNA expression), and EREG-EXPss (Epigenetically regulated RNA expression) (19).

### Analysis of tumor immune microenvironment

The R software package “ESTIMATE” was used to calculate stromal, immune, and ESTIMATE scores for patients in each tumor based on gene expression (20). In addition, the ImmuneScore, StromaScore and MicroenvironmentScore for patients in each tumor were evaluated using the xCell algorithm (21). The CIBERSOR algorithm was utilized to analyze the infiltration score of 22 immune cells for patients in each tumor (22). Furthermore, the MHC, EC, SC, CP, AZ and IPS Immunophenoscores for patients in each tumor were evaluated based on gene expression using the IPS algorithm (23).

### Real-time quantitative PCR (RT-qPCR)

RT-qPCR was used for RNAi validation and gene expression analysis. Total RNA was isolated using Trizol (Thermo Fisher), followed by reverse transcription into complementary DNA (cDNA) using NovoScript^®^ Plus All-in-one 1st Strand cDNA Synthesis SuperMix (gDNA Purge) (novoprotein E047-01B) as per the manufacturer’s instructions. Subsequently, the cDNA served as a template for RT-qPCR analysis using NovoStart^®^ SYBR qPCR SuperMix Plus (novoprotein E096-01A). Using GAPDH as an internal control, relative gene expression levels were assessed using the 2^−ΔΔCT^ method. All primer sequences are listed in **Supplementary Table 1**.

### Western blot

In brief, protein extracts were prepared using RIPA lysis buffer (Epizyme) supplemented with a 100x EDTA-free Protease Inhibitor Cocktail (Epizyme). The cell lysates were separated by electrophoresis and transferred to polyvinylidene difluoride (PVDF) membranes (Merck Millipore) via electroblotting. The membranes were blocked with 5% non-fat milk in TBS for 2 hours at room temperature, then incubated overnight at 4°C with primary antibodies: anti-TSPAN4 (sigma, SAB2106687), anti-GFP (proteintech, 66002-1-Ig), anti-β-Actin (proteintech, 66009-1-Ig) or anti-GAPDH (bioworld, PA5-69344). After washing the blots three times with TBST, they were incubated for 2 hours at room temperature with HRP-conjugated AffiniPure Goat Anti-Rabbit IgG (proteintech, SA00001-2) or HRP-conjugated AffiniPure Goat Anti-Mouse IgG (proteintech, SA00001-1). Finally, the labeled proteins were detected using the ECL reagent.

### Assessment of cell proliferation

Glioma cells were subjected to TSPAN4 knockdown or overexpression via infection with specific adenoviruses. Cell proliferation was assessed using the Cell Counting Kit-8 (CCK-8, Dojindo) and the EdU Kit (Beyotime Biotecnology) as per the manufacturer’s instructions. For the CCK-8 assay, 100 μl of cell suspension (5000 cells/well) was added to each well of a 96-well plate and incubated for 24 hours in a humidified incubator. Following the appropriate incubation period (e.g., 24, 48, or 72 hours), 10 μl of CCK-8 solution was added to each well and further incubated for 1 - 4 hours. Absorbance at 450 nm was then measured using a microplate reader. For the EdU assay, TSPAN4-deficient and control glioma cells were cultured in glass-bottomed dishes. Following a 24-hour incubation, cells were exposed to EdU solution and cultured for 2 hours under optimal conditions. Subsequently, cells were treated with fixation solution for 15 minutes and permeabilization buffer for 20 minutes. The reaction mixture was applied to fluorescently label EdU for 30 minutes, followed by staining with DAPI. Cell analysis was conducted using a fluorescence microscope.

### Migrasome staining and detection

Fluorescently tagged wheat-germ agglutinin (WGA), a lectin that specifically binds to sialic acid and N-acetyl-D-glucosamine, was employed to label migrasomes in living cells (24). Consequently, we utilized fluorescently tagged WGA as a probe for migrasome identification. U87 MG and LN229 cells were stained with 1 μg/ml WGA-FITC (Sigma-Aldrich, L4895), which was present in the medium, followed by image capture.

### Macrophage polarization assay

Plates were seeded with THP-1 suspension cells, induced to differentiate into mature macrophages with PMA (final concentration 100 ng/ml). Following 48 hours of culture, the cells transitioned from suspension to adherence. Subsequently, treated macrophages were co-cultured with control glioma cells or cells with TSPAN4 knockdown or overexpression for 48 hours. Following co-culture, M1 and M2 macrophage markers were assessed using flow cytometry and RT-qPCR.

### Statistical analysis

The Wilcoxon test was used to assess the significance of gene expression differences between tumors and normal tissues in the pan-cancer dataset. One-way ANOVA was employed to assess differences among multiple clinical stages of the samples. The significance of differences in promoter DNA methylation was assessed using the Student’s t-test. The significance of prognostic differences among different groups was assessed using the log-rank test. Statistical analyses were conducted using R software (version 4.2.0) and GraphPad Prism 9.0, with statistical significance set at p < 0.05.

## Results

### DNA methylation regulates TSPAN4 expression in pan-cancer

**Figure 1:**
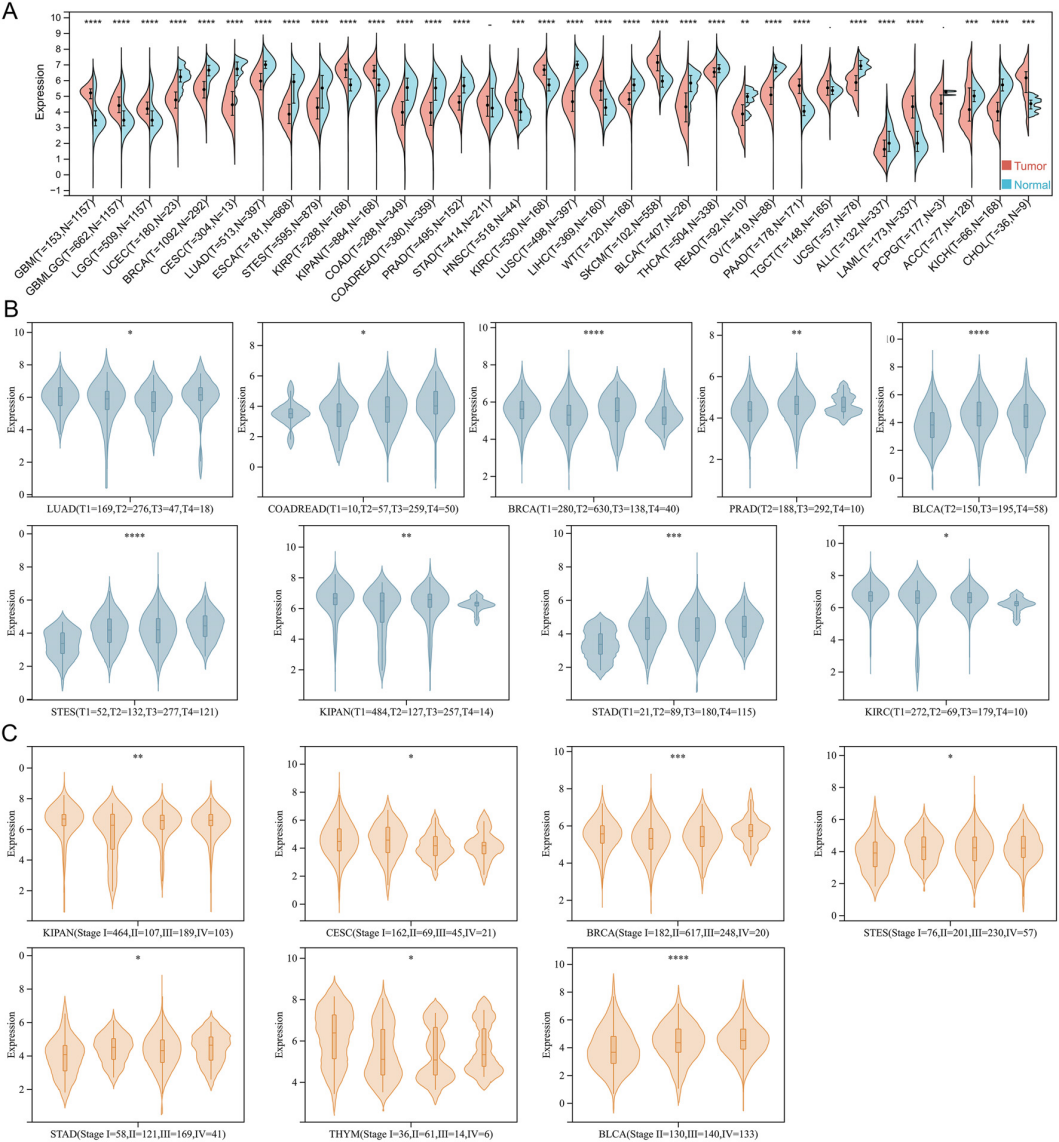
The expression of TSPAN4 in pan-cancer. **(A)** Split violin diagram showing TSPAN4 expression between normal and tumor samples in the TCGA TARGET GTEx dataset. **(B)** Violin plot showing TSPAN4 expression between different T staging subgroups. **(C)** Violin plot showing TSPAN4 expression between different stages. *P< 0.05; **P< 0.01; ***P< 0.001, ****P< 0.0001.

TSPAN4 is both a visual marker migrasome and a key regulator for migrasome formation. However, its role in tumorigenesis and progression remains unclear. Therefore, we analyzed the expression of TSPAN4 in the pan-cancer dataset (TCGA TARGET GTEx) (**Figure 1A**). With the exceptions of STAD, TGCT, and PCPG, TSPAN4 exhibited aberrant expression across all tumor tissues relative to their corresponding normal tissues, with up-regulation in 12 tumors and down-regulation in 19 tumors (**Figure 1A**). Additionally, we assessed TSPAN4 expression across various TNM-staging groups within each tumor type, revealing significant differences in T-staging among LUAD, COADRAD, BRCA, STES, KIPAN, STAD, PRAD, KIRC, and BLCA (**Figure 1B**). Furthermore, TSPAN4 exhibited differential expression across different stages of CESC, BRCA, STES, KIPAN, STAD, THYM, and BLCA (**Figure 1C**).

**Figure 2:**
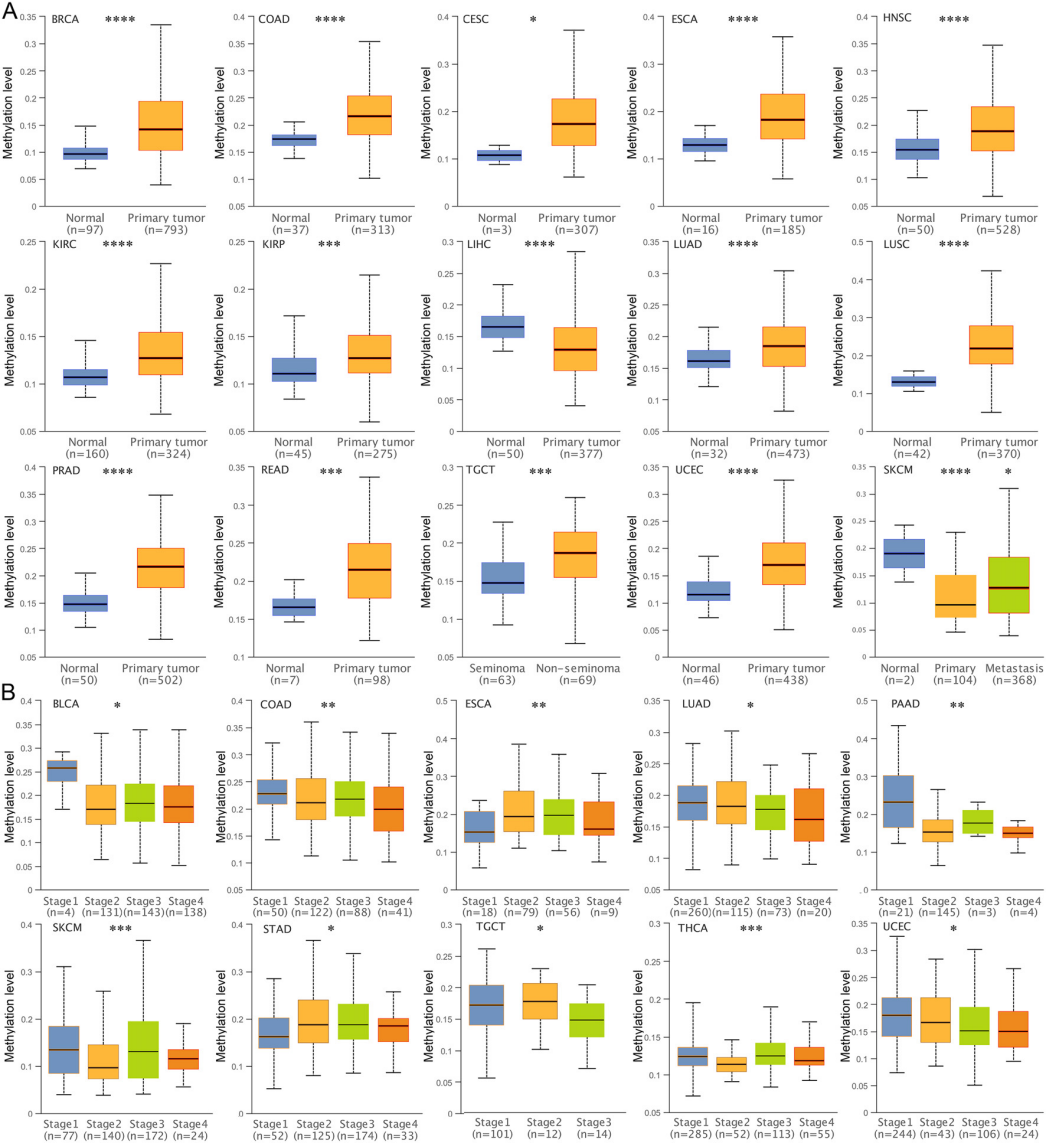
Analysis of DNA methylation level of TSPAN4 promoter in pan-cancer. **(A)** Box-whisker plot showing the DNA methylation level (average beta value) of TSPAN4 promoter in pan-cancer dataset. **(B)** Box-whisker plot showing the DNA methylation level (average beta value) between different stages in pan-cancer dataset. *P< 0.05; **P< 0.01; ***P< 0.001, ****P< 0.0001.

DNA methylation is an important epigenetic regulatory mechanism that leads to chromatin condensation and transcriptional repression. Therefore, we analyzed the DNA methylation status of the TSPAN4 promoter. The methylation level of 12 tumors (BRCA, COAD, CESC, ESCA, HNSC, KIRC, KIRP, LUAD, LUSC, PRAD, READ, TGCT, UCEC) was higher than that of normal tissues, and two tumors (LIHC, SKCM) had lower methylation levels than normal tissues (**Figure 2A**). Alterations in TSPAN4 promoter methylation were consistent with changes in TSPAN4 expression in BRCA, COAD, CESC, ESCA, LUAD, LUSC, PRAD, READ, UCEC, LIHC, and SKCM (**Figures 1A**, **2A**). Moreover, the methylation level of the TSPAN4 promoter exhibited significant variation across different stages of BLCA, COAD, ESCA, LUAD, PAAD, SKCM, STAD, TGCT, THCA, and UCEC (**Figure 2B**). Thus, altered DNA methylation might underlie the aberrant expression of TSPAN4 in pan-cancer.

### Prognostic value of TSPAN4 expression in pan-cancer

**Figure 3:**
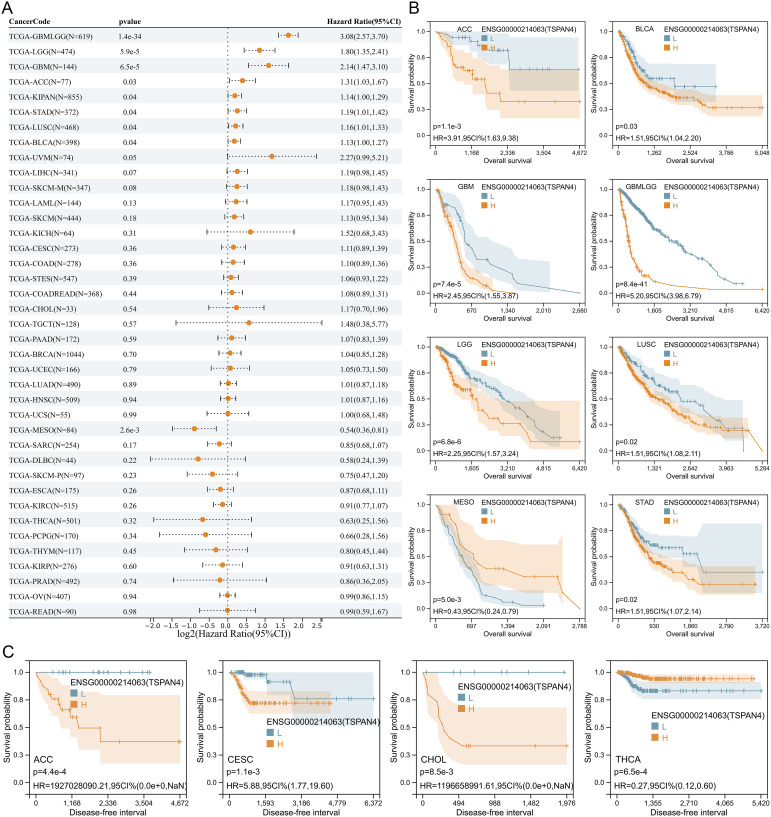
Prognostic analysis of TSPAN4 in pan-cancer dataset. **(A)** Cox regression analysis of TSPAN4 in pan-cancer dataset. **(B)** Kaplan-Meier curve describing the overall survival of the TSPAN4 high-expression group and low-expression group in pan-cancer. **(C)** Kaplan-Meier curve describing the Disease-free survival of the TSPAN4 high-expression group and low-expression group in pan-cancer.

Cox regression analysis revealed that TSPAN4 acted as a risk factor in GBM, LGG, GBMLGG, ACC, KIPAN, STAD, LUSC, and BLCA, while exhibiting protective effects in MESO (**Figure 3A**). Likewise, high TSPAN4 expression correlated with worse overall survival in GBM, LGG, GBMLGG, ACC, STAD, LUSC, and BLCA, whereas in MESO, high expression corresponded to improved overall survival compared with low expression (**Figure 3B**). Kaplan-Meier analysis showed a significant disparity in progression-free survival between the high and low TSPAN4 expression groups in CESC, ACC, CHOL, and THCA (**Figure 3C**). Hence, further investigation into the role of TSPAN4 in GBM, LGG, GBMLGG, ACC, KIPAN, STAD, LUSC, and BLCA is warranted.

### Correlations between TSPAN4 and tumor heterogeneity, stemness in pan-cancer

**Figure 4:**
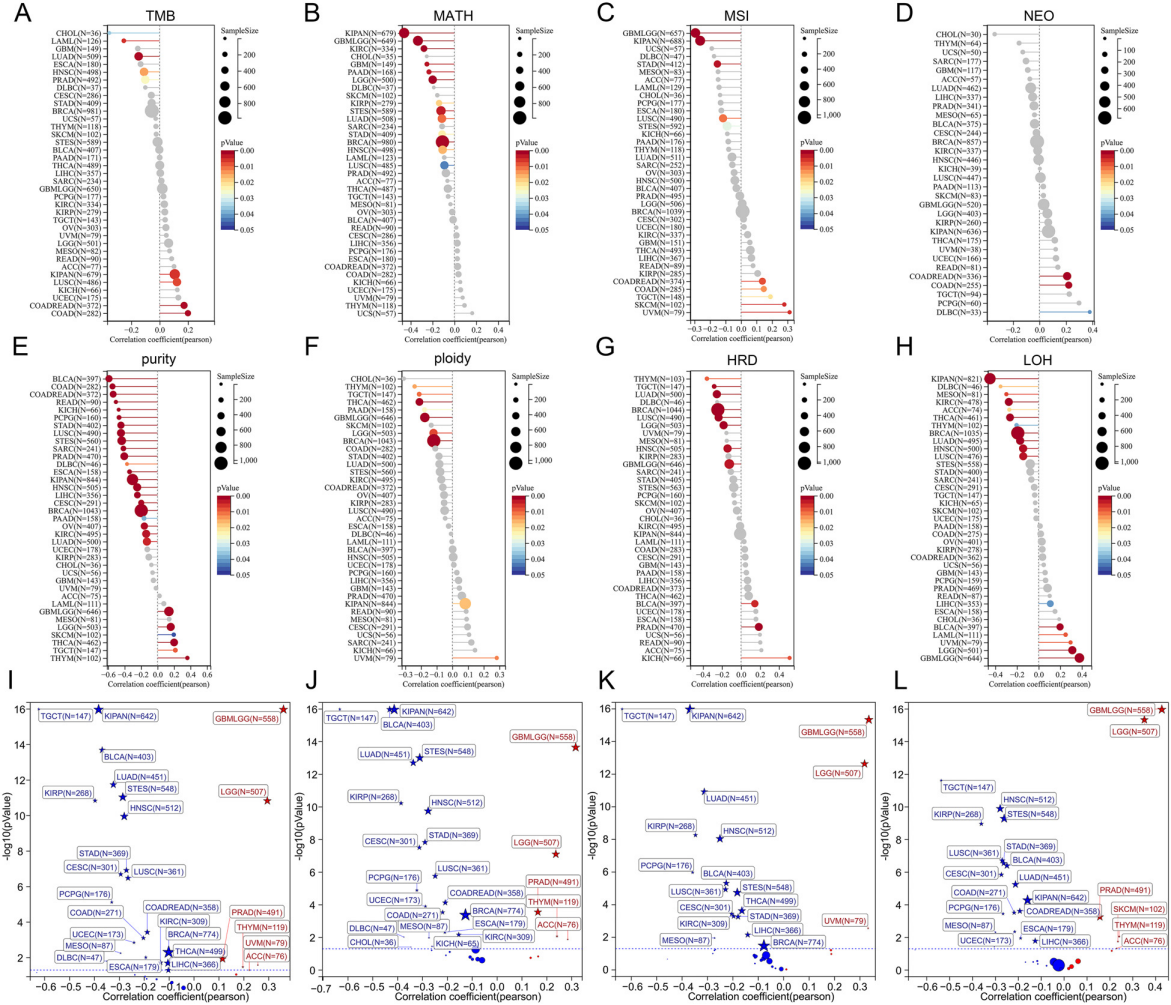
Analysis of the relationship between TSPAN4 and tumor heterogeneity and stemness. **(A)** The relationship between TSAPN4 and TMB, **(B)** MATH, **(C)** MSI, **(D)** NEO, **(E)** purity, **(F)** ploidy, **(G)** HRD, **(H)** LOF in the pan-cancer dataset. **(I)** The relationship between TSAPN4 and DNAss, **(J)** EREG-METHs, **(K)** DMPss, and **(L)** ENHs in the pan-cancer dataset.

Heterogeneity is a distinguishing feature of tumors that can predict treatment response and prognosis. Herein, we calculated correlations between TSPAN4 expression and tumor genomic heterogeneity in pan-cancer. Significant associations were observed: 9 tumors with TMB, 10 tumors with MSI, 13 tumors with MATH, 3 tumors with NEO, 28 tumors with purity, 9 tumors with ploidy, 11 tumors with HRD, and 17 tumors with LOH (**Figures 4A–H**). In tumors where TSPAN4 expression correlated with prognosis, particularly in GBMLGG and LUSC, TSPAN4 exhibited a strong association with genomic heterogeneity (**Figures 4A–H**).

Additionally, we calculated the stemness scores in pan-cancer. Correlation analysis showed that TSPAN4 correlated with DNAss in 27 tumors (**Figure 4I**), with EREG-METHs in 27 tumors (**Figure 4J**), with DMPss in 19 tumors (**Figure 4K**), and with ENHss in 24 tumors (**Figure 4L**). For tumors associated with TSPAN4 prognosis, stemness scores were positively correlated with TSPAN4 in GBMLGG, LGG, and ACC, while being negatively associated in STAD, LUSC, MESO, and BLCA (**Figures 4I–L**).

### Investigating the correlation between TSPAN4 expression and the tumor immune microenvironment

**Figure 5:**
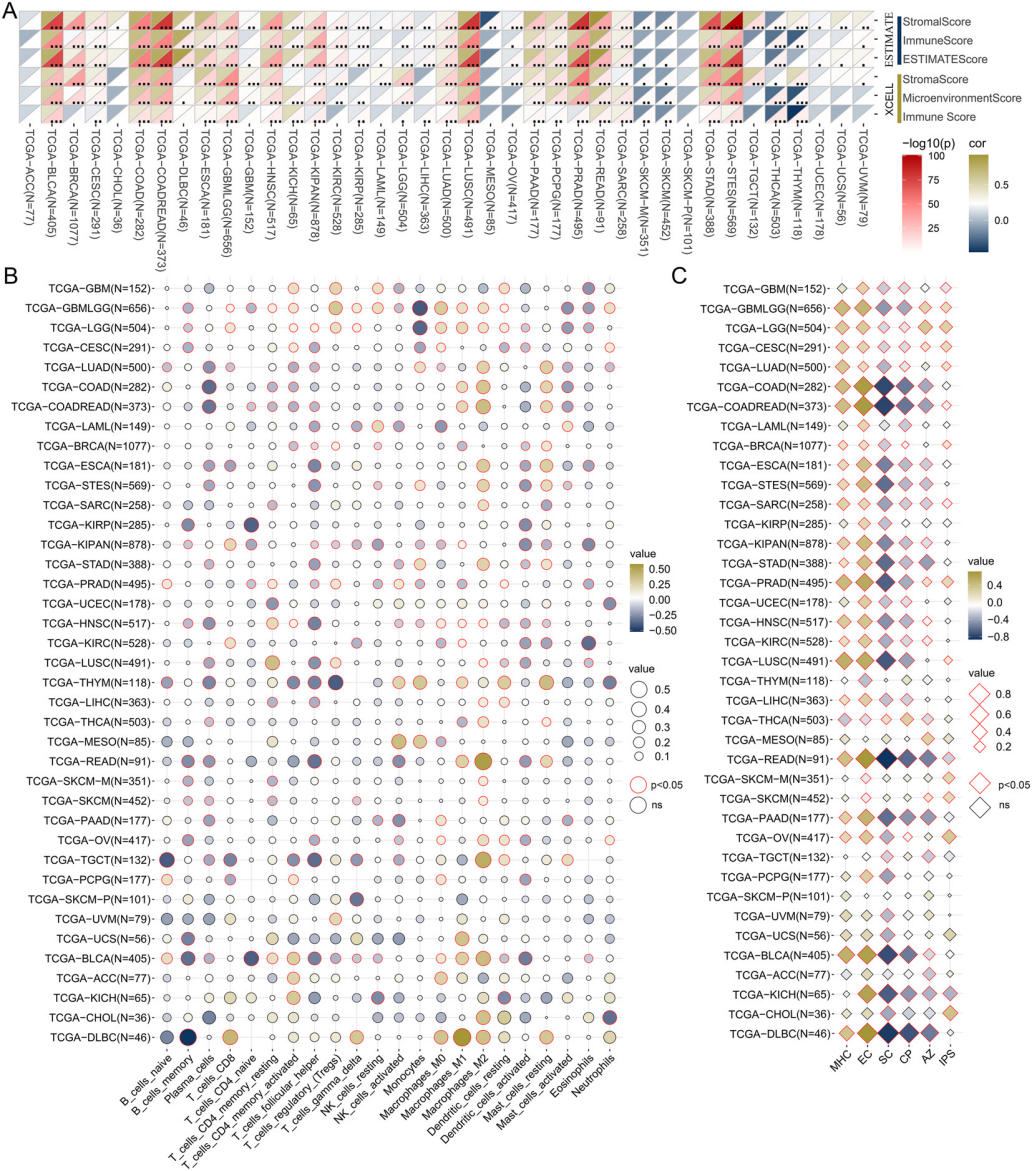
Analysis of the relationship between TSPAN4 and tumor microenvironment in pan-cancer dataset. **(A)** The relationship between TSPAN4 and microenvironment scores calculated by ESTIMATE and XCELL algorithm. **(B)** The relationship between TSPAN4 and 22 infiltrating immune cells calculated by CIBERSORT algorithm. **(C)** The relationship between TSPAN4 and Immunophenoscore calculated by IPS algorithm. *, P< 0.05; **, P< 0.01; ***, P< 0.001.

Subsequently, we examined the association between TSPAN4 expression and the tumor microenvironment, infiltrating immune cells, and immunophenotype. Tumor microenvironment scores for pan-cancers were calculated by ESTIMATE and XCELL algorithm (**Figure 5A**). Focusing on tumors influenced by TSPAN4, we found that the microenvironment scores were positively associated with TSPAN4 expression in BLCA, GBMLGG, LUSC, and STAD, but insignificantly in ACC and MESO (**Figure 5A**). In addition, twenty-two tumor-infiltrating immune cells across pan-cancer were calculated using the CIBERSORT algorithm (**Figure 5B**). We observed that TSPAN4 expression correlated with the level of immune cell infiltration in BLCA, GBMLGG, LUSC and STAD, but only a few significant correlations in ACC and MESO (**Figure 5B**). TSPAN4 expression was positively correlated with M2 Macrophages in BLCA, GBMLGG, LUSC and STAD, and positively correlated with T cells regulatory (Tregs) in GBMLGG and LUSC (**Figure 5B**). We then employed the IPS algorithm to analyze the immunophenotype and found that TSPAN4 was correlated with MHC, EC, SC, CP and AZ immunophenoscore in BLCA, GBMLGG, LUSC and STAD, and with IPS immunophenoscore in GBMLGG and LUSC (**Figure 5C**).

**Figure 6:**
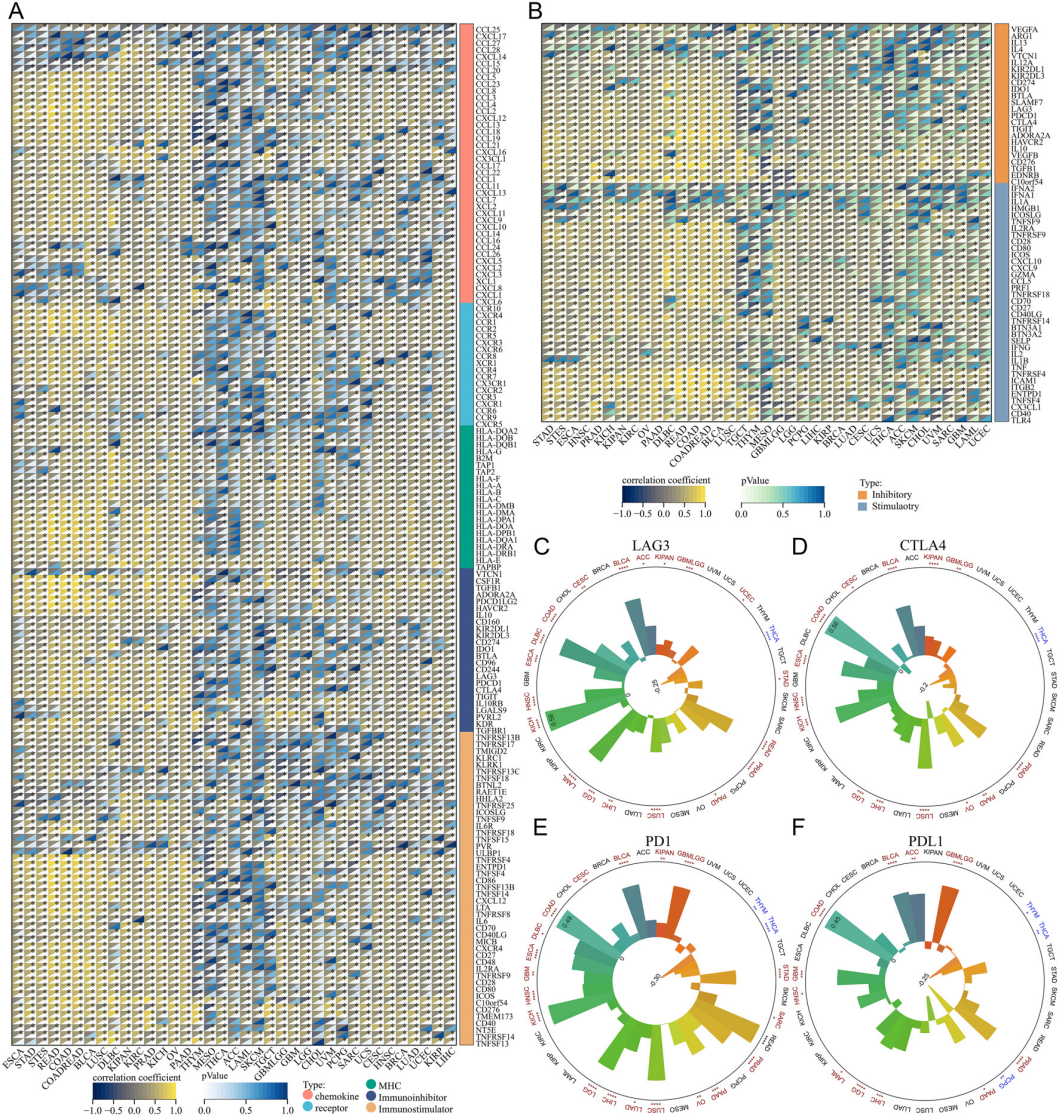
Analysis of the relationship between TSPAN4 and tumor immune characteristics in the pan-cancer dataset. **(A, B)** Correlation between TSPAN4 and immunomodulators in the pan-cancer dataset. **(C)** Correlation between TSPAN4 and four immune checkpoints LAG3, **(D)** CTLA4, **(E)** PD1 and **(F)** PDL1 in pan-cancer dataset. *P< 0.05; **P< 0.01; ***P< 0.001, ****P< 0.0001.

Moreover, we compiled five immunoregulatory signatures (chemokine, receptor, MHC, Immunoinhibitor, Immunostimulator) (25) and two immune checkpoint signatures (Inhibitory and Stimulatory) (18) from prior research (**Supplementary Tables 2**, **3**). TSPAN4 exhibited associations with most immunomodulators in BLCA, GBMLGG, KIPAN, LUSC, and STAD; nevertheless, there were few significant associations in ACC and MESO (**Figures 6A, B**). Furthermore, we observed a significant association between TSPAN4 expression and well-known immune checkpoints, including PD-L1, PD-1, CTLA-4, and LAG-3 in BLCA, GBMLGG, KIPAN, and LUSC (**Figures 6C–F**).

### Comprehensive analysis of TSPAN4 in glioma

**Figure 7:**
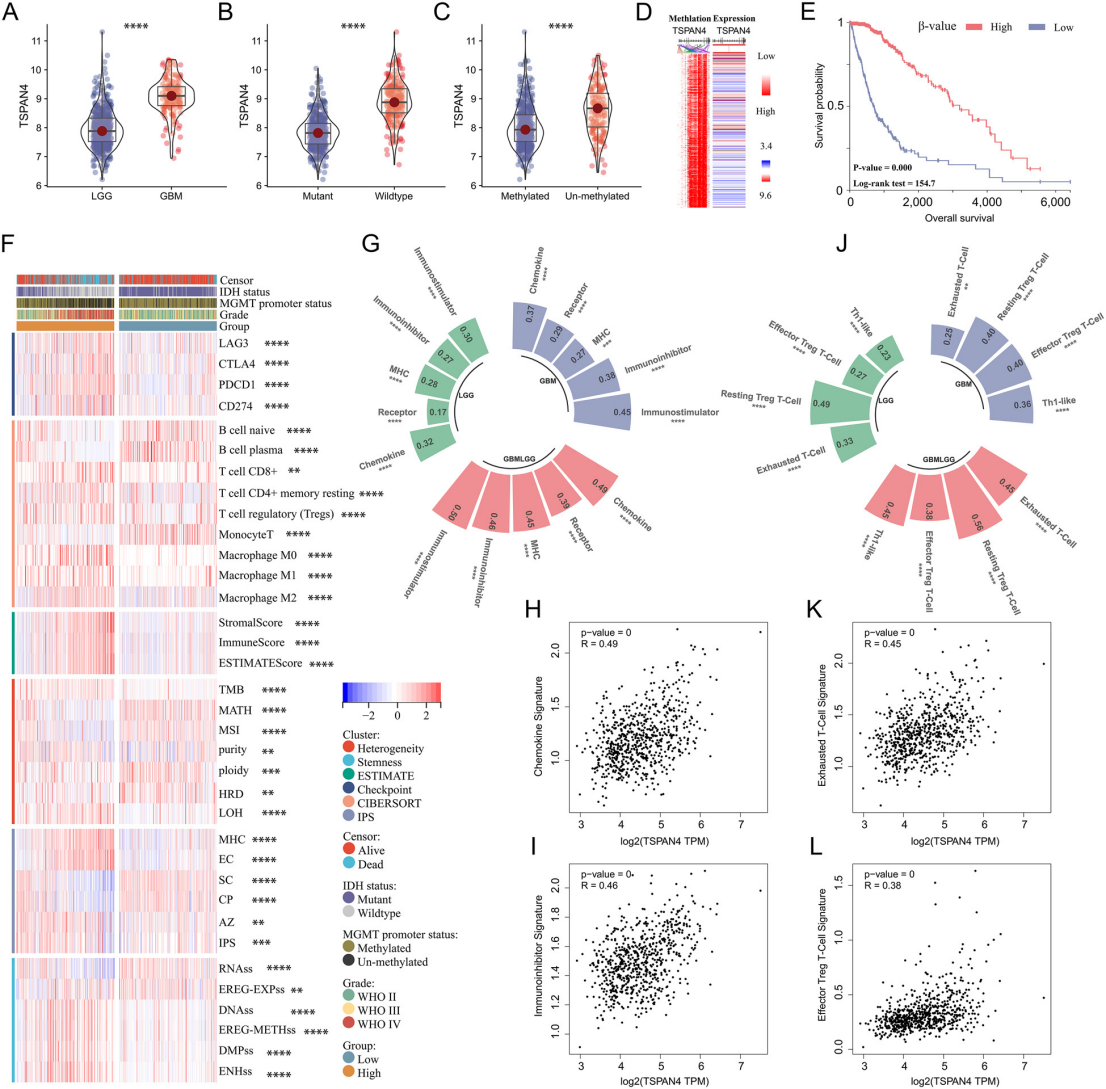
Comprehensive analysis of TSPAN4 in glioma. **(A-C)** Violin plots displaying TSPAN4 expression level in subgroups with different clinical characteristics in TCGA GBMLGG cohort. **(D)** Heatmap displaying the relationship in TSPAN4 expression and methylation levels. **(E)** Kaplan-Meier curve describing the overall survival of TSPAN4 high-methylation group and low-methylation group in TCGA GBMLGG cohort. **(F)** Heatmap displaying the differences in heterogeneity, stemness and immune status between TSPAN4 high-expression and low-expression groups. **(G-I)** Correlation between TSPAN4 and immunoregulatory signatures in glioma. **(J-L)** Correlation between TSPAN4 and immunocyte signatures in glioma. **P< 0.01; ***P< 0.001, ****P< 0.0001.

Given the extreme correlation between TSPAN4 and glioma characteristics, we further analyzed its role in the TCGA GBMLGG cohort. TSPAN4 was upregulated in GBM, IDH wild-type and MGMT unmethylated subgroups, respectively, compared with LGG, IDH mutant, and MGMT methylated subgroups (**Figures 7A–C**). Furthermore, our results indicated that TSPAN4 expression and methylation levels were mutually exclusive, and patients with higher TSPAN4 methylation levels had worse survival prognosis (**Figures 7D, E**). Similarly, differences in heterogeneity, stemness, and immunological status were observed between the TSPAN4 high- and low-expression groups, consistent with the findings in the previous section (**Figure 7F**). For example, the expression of the checkpoints LAG3, CTLA4, PD1 and PDL1 was increased in the TSPAN4 high-expression group; the number of macrophages M2 was also increased (**Figure 7F**). As mentioned earlier, TSPAN4 expression correlated with immunoregulatory signatures, including chemokine, receptor, MHC, Immunoinhibitor, and Immunostimulator, in glioma (**Figures 7G–I**). In addition, we found that TSPAN4 expression was significantly associated with immunosuppressive cell-related signatures, including exhausted T-cell, effector Treg T-cell, resting Treg T-cell and Th1-like cell (**Figures 7J–L**).

**Figure 8:**
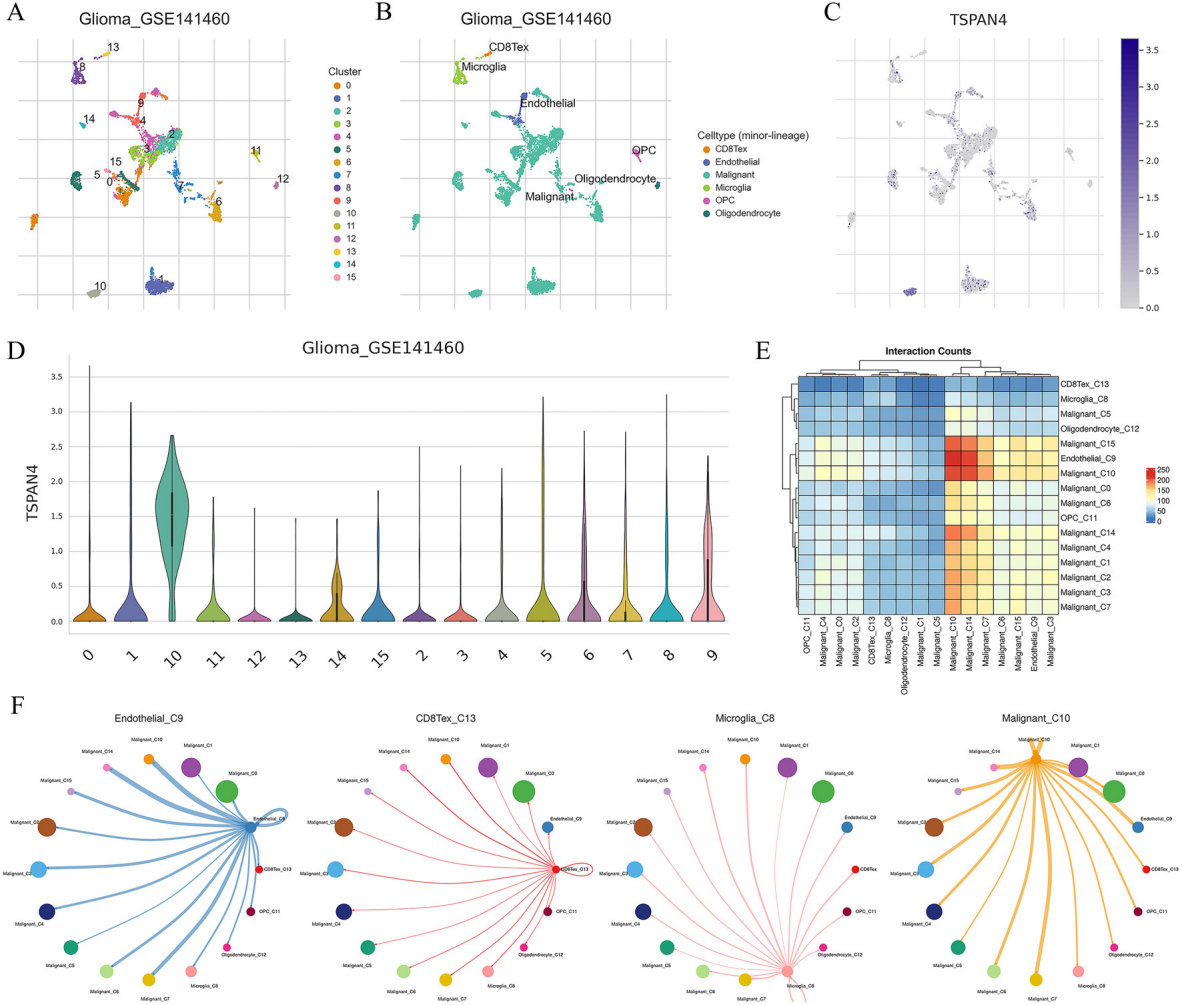
Analysis of TSPAN4 in the GBM single-cell RNA-seq dataset: **(A)** UMAP analysis of all cells in GSE141460. **(B)** UMAP plot displaying cell type annotation. **(C)** Expression of TSPAN4 across cell lineages depicted in UMAP plots. **(D)** Expression of TSPAN4 across cell lineages illustrated in violin plots. **(E)** Heatmap demonstrating ligand-receptor interactions among cell clusters. **(F)** Circos plot illustrating ligand-receptor interactions between malignant cluster 10 and other cell types.

Further analysis of TSPAN4 was conducted using the GBM single-cell RNA-seq dataset (GSE141460). Uniform manifold approximation and projection (UMAP) analysis revealed 16 unsupervised clusters across all cells (**Figure 8A**), and cell type annotation revealed 8 cell types: CD8Tex, endothelial, malignant, microglia, OPC, and oligodendrocyte (**Figure 8B**). We then explored TSPAN4 expression across cell lineages and identified high expression in malignant cluster 10 (**Figures 8C, D**). Subsequently, we analyzed the cell-cell interaction (CCI) network and identified significant interactions between malignant cluster 10 and CD8Tex, microglia, and endothelial (**Figures 8E, F**), suggesting an association between the TSPAN4 high-expression subpopulation and the tumor immune microenvironment.

### TSPAN4 regulates extracellular matrix and immune-related pathways in glioma

**Figure 9:**
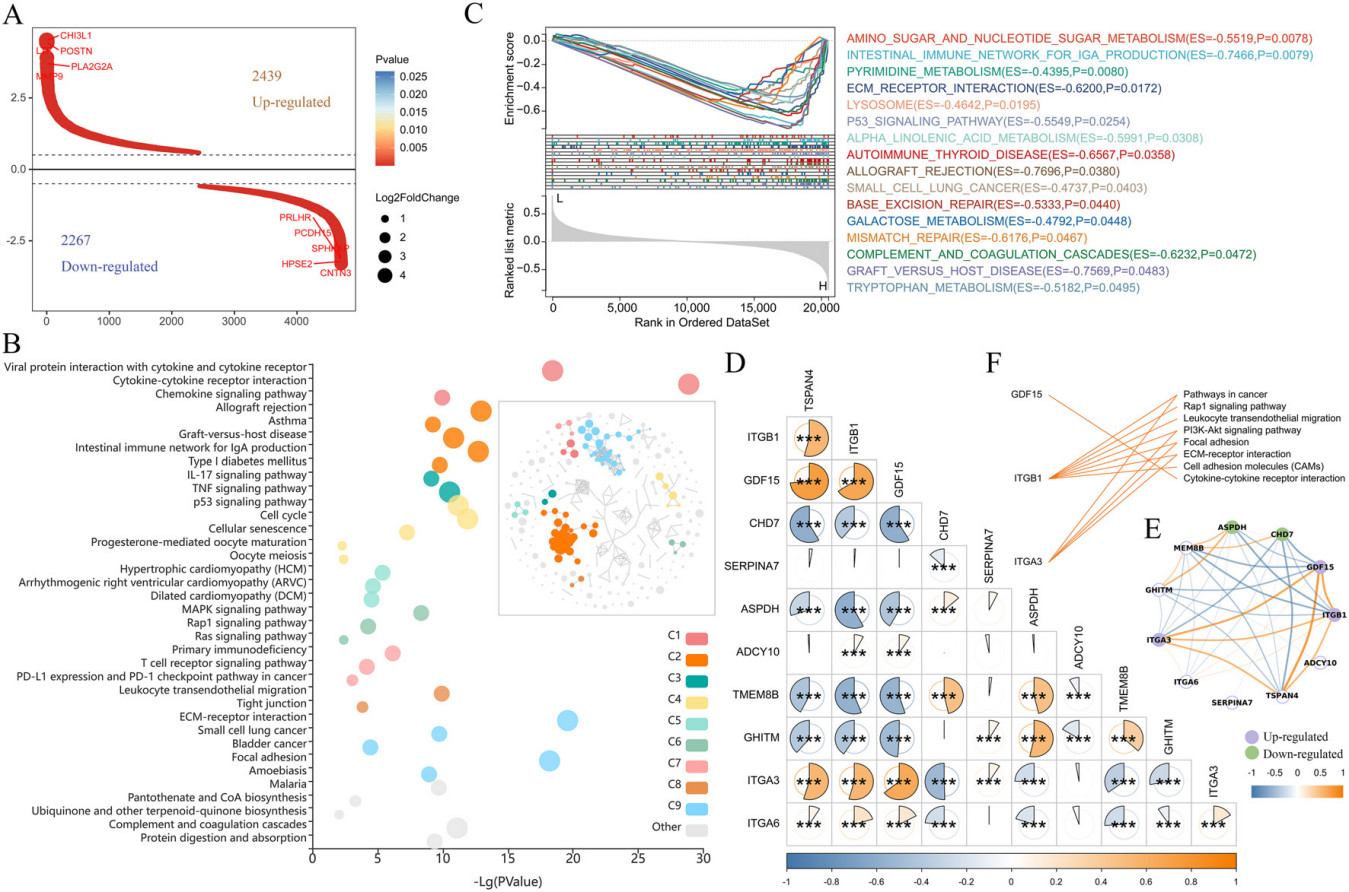
Pathway analysis regulated by TSPAN4 in gliomas. **(A)** Screening of differentially expressed genes between TSPAN4 high- and low-expression groups. **(B)** KEGG pathway analysis of up-regulated genes, C1-C9 represents different clusters of signaling pathways. **(C)** GSEA of TSPAN4 in TCGA cohort. **(D, E)** Correlation analysis between TSAPN4 and its interacting partners obtained from the STRING database. **(F)** Line link showing the KEGG pathways involved by the up-regulated partners. *P< 0.05, ***P< 0.001.

Subsequently, we analyzed the signaling pathways in which TSPAN4 may be involved in glioma. We identified 4706 differentially expressed genes (|foldchange|≥1.5, P<0.05) between the TSPAN4 high- and low-expression groups, of which 2439 genes were up-regulated and 2267 genes were down-regulated in the high-expression group (**Figure 9A**). KEGG analysis showed that up-regulated genes were enriched in immune-related signaling pathways, including cytokine-cytokine receptor interaction, leukocyte transendothelial migration, chemokine signaling pathway, PD-L1 expression and PD-1 checkpoint pathway in cancer, as well as extracellular matrix and adhesion related pathways, such as ECM-receptor interaction, focal adhesion, and cell adhesion molecules (CAMs) (**Figure 9B**). Moreover, gene set enrichment analysis (GSEA) indicated that samples in the high-expression group exhibited enrichment in gene features associated with metabolism, ECM receptor interaction, and the P53 signaling pathway compared with the low-expression group (**Figure 9C**).

Ten predicted functional partners of TSPAN4 were obtained from the STRING database, among which ITGB1, GDF15 and ITGA3 showed potential co-expression with TSPAN4. Correlation analysis revealed that TSPAN4 was positively correlated with ITGB1, GDF15, ITGA3 and ITGA6, and negatively correlated with CHD7, ASPDH, TMEM8B and GHITM in glioma (**Figures 9D, E**). Compared with the TSPAN4 low-expression group, ITGB1, GDF15 and ITGA3 were significantly up-regulated in the high-expression group (**Figure 9E**) and participated in immune-related, ECM and adhesion-related pathways (**Figure 9F**).

### TSPAN4 facilitates glioma cell proliferation, migrasome formation, and induces M2-type polarization of macrophages

Additionally, we downloaded the mRNAseq_325 and mRNAseq_693 cohorts to comprehensively analyze TSPAN4 expression across subtypes and confirm its prognostic value. The results showed that TSPAN4 was up-regulated in high grade glioma compared with low grade glioma in mRNAseq_325 and mRNAseq_693 cohorts (**Supplementary Figures 1A, B**). And TSPAN4 expression in IDH wild-type, MGMT un-methylated, and old subtypes was higher than the corresponding subtypes (**Supplementary Figures 1A, B**). Kaplan-Meier showed that in the mRNAseq_325 and mRNAseq_693 cohorts, the overall survival of the TSPAN4 high expression group was shorter than that of the low expression group (**Supplementary Figures 1C, D**).

**Figure 10:**
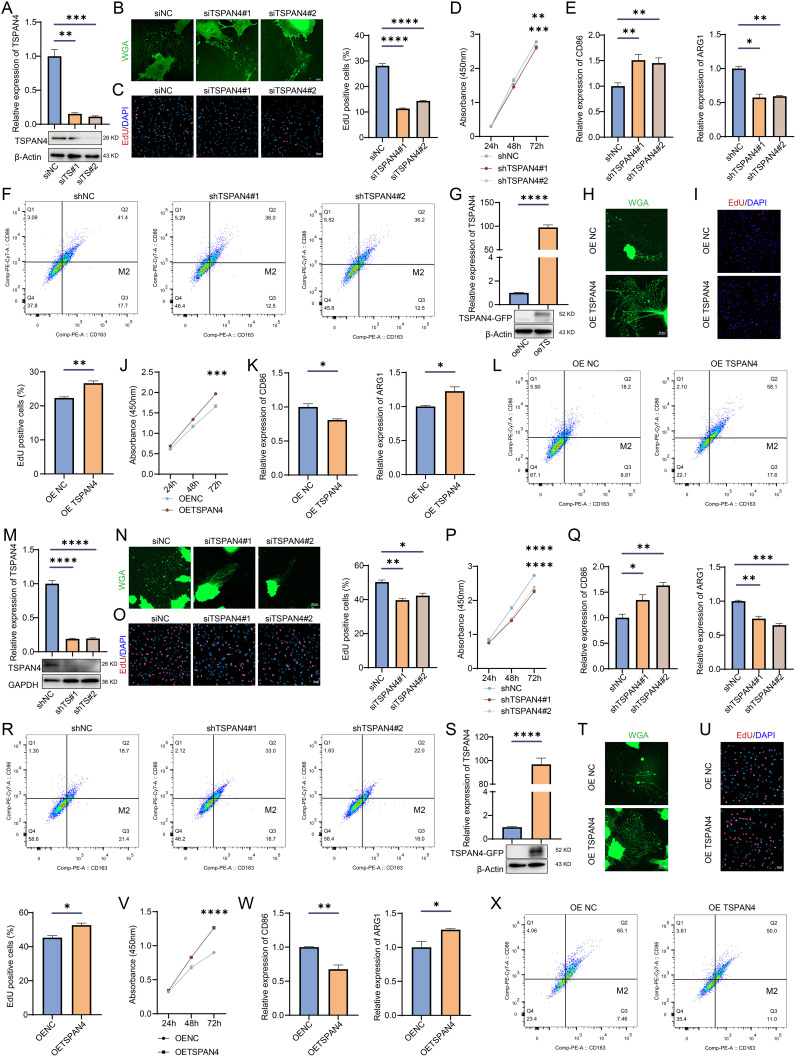
Effect of TSPAN4 on glioma cells. **(A)** Confirmation of TSPAN4 knockdown efficiency in U87 MG cells **(B)** Representative images of migrasomes in TSPAN4 knockdown and control U87 MG cells. **(C)** EdU assay and **(D)** CCK8 assay for assessing the proliferative capacity of TSPAN4 knockdown and control U87 MG cells. **(E)** qRT-PCR and **(F)** flow cytometry analyses to evaluate M1/M2 macrophage polarization. **(G)** Validation of TSPAN4 overexpression in U87 MG cells. **(H)** Representative images of migrasomes in TSPAN4 overexpression and control U87 MG cells. **(I)** EdU assay and **(J)** CCK8 assay to evaluate cell proliferation ability of TSPAN4 overexpression and control U87 MG cells. **(K)** qRT-PCR and **(L)** flow cytometry analyses of M1/M2 macrophage polarization. **(M)** Verification of TSPAN4 knockdown efficiency in LN229 glioma cells **(N)** Representative images of migrasomes in TSPAN4 knockdown and control LN229 glioma cells. **(O)** EdU assay and **(P)** CCK8 assay to evaluate cell proliferation ability of TSPAN4 knockdown and control LN229 glioma cells. **(Q)** qRT-PCR and **(R)** flow cytometry analyses of M1/M2 macrophage polarization. **(S)** Validation of TSPAN4 overexpression in LN229 glioma cells. **(T)** Representative images of migrasomes in TSPAN4 overexpression and control LN229 glioma cells. **(U)** EdU assay and **(V)** CCK8 assay to evaluate cell proliferation ability of TSPAN4 overexpression and control LN229 glioma cells. **(W)** qRT-PCR and **(X)** flow cytometry analyses of M1/M2 macrophage polarization. *P< 0.05; **P< 0.01; ***P< 0.001, ****P< 0.0001.

To investigate the role of TSPAN4 in glioma cells, we knocked down TSPAN4 in U87 MG glioma cells (**Figure 10A**). This knockdown significantly inhibited migrasome formation (**Figure 10B**) and reduced the proliferative capacity of TSPAN4-deficient U87 MG cells (**Figures 10C, D**). In co-culture experiments, we further confirmed that TSPAN4 knockdown impeded macrophage polarization toward the M2 phenotype (**Figures 10E, F**). Conversely, TSPAN4 overexpression in U87 MG cells promoted migrasome formation (**Figures 10G, H**), enhanced proliferation (**Figures 10I, J**), and facilitated M2 polarization of macrophages (**Figures 10K, L**). We validated these findings in LN229 glioma cells as well. TSPAN4 knockdown in LN229 cells suppressed migrasome formation (**Figures 10M, N**), decreased proliferation (**Figures 10O, P**), and inhibited macrophage polarization toward M2 (**Figures 10Q, R**). In contrast, TSPAN4 overexpression in LN229 cells enhanced migrasome formation (**Figures 10S, T**), proliferation (**Figures 10U, V**), and promoted M2 macrophage polarization (**Figures 10W, X**).

## Conclusions

Our study offers comprehensive insights into the role of TSPAN4 in cancer biology, highlighting its potential as a prognostic marker across diverse cancer types, along with its significance as a target for immunotherapy.

## Discussion

TSPAN4 serves a dual role within migrasomes: it acts as both a marker for their visualization and a crucial regulator of their formation. Our analysis of the pan-cancer dataset unveiled aberrant expression patterns of TSPAN4 across various tumor types, highlighting its role as a prognostic factor in several cancers. Notably, we observed alterations in DNA methylation levels of the TSPAN4 promoter region, which were associated with its dysregulated expression in pan-cancer (**Figure 2**), indicating a potential epigenetic regulatory mechanism underlying TSPAN4 dysregulation.

Information transfer between human cells commonly occurs via direct contact with neighboring cells; however, it is more prevalent for cells to secrete a variety of chemicals or extracellular vesicles (EVs) to regulate their own metabolism and function as well as that of other cells. EVs consist of a wide array of distinct particles, including exosomes, large oncosomes (LOs), apoptotic bodies, ARMMs, and other small exosome-sized EVs, microvesicles (MVs) (26–28). EVs, originating from various cells, can carry a variety of substances like DNA, RNA, proteins, lipids, and metabolites, and can be internalized by other cells through various mechanisms, including endocytosis, receptor-ligand interactions, or fusion with cell membranes, both locally by neighboring cells and distantly after transfer to the peripheral circulation (29, 30). It is now recognized that cells release EVs as part of normal physiological processes. However, under pathophysiological conditions, especially during cancer progression, this process can be exploited (29, 31, 32).

Migrasomes display notable distinctions from EVs such as exosomes, microvesicles, and apoptotic bodies in terms of size, composition, release mechanism, and life cycle. Nonetheless, they share certain physical and behavioral traits with EVs, including the ability of shed vesicles to be absorbed by neighboring and distal cells (33, 34). Migrasomes represent a burgeoning area of biological research with the potential to revolutionize our comprehension of cell communication. Moreover, they hold significant promise for disease diagnostics and therapeutics. However, it remains uncertain whether migrasomes play a role in mediating communication between tumor cells and the microenvironment, as well as in facilitating long-distance communication between tumor cells and metastatic target organs.

Upon rupture, migrasomes release a multitude of factors into the surrounding environment. As expected, our analysis uncovered a correlation between TSPAN4 expression the tumor immune microenvironment. Importantly, TSPAN4 expression correlated with immunoregulatory and immune checkpoint molecules, and it regulated immune cell infiltration and modulated immune cell function within the tumor microenvironment. These findings suggest that TSPAN4 may contribute to immune evasion, making it a potential target for immunotherapy in certain cancers. Additionally, in tumors like GBMLGG and LUSC, TSPAN4 exhibited significant associations with genomic heterogeneity, underscoring its potential impact on tumor diversity and evolution. Furthermore, our functional analysis demonstrated the influence of TSPAN4 on glioma cell proliferation, migrasome formation, and macrophage polarization, illustrating its multifaceted involvement in tumor progression and immune modulation. However, whether TSPAN4 exerts a similar role in other tumor types, particularly in LUSC, remains unclear and requires further experimental validation.

## Data Availability

The original contributions presented in the study are included in the article/**Supplementary Material**. Further inquiries can be directed to the corresponding author.

## Supplementary material

Supplementary Table 1 Primers and siRNAs used in this study.

Supplementary Table 2 Gene list of five immunoregulatory signatures (chemokine, receptor, MHC, Immunoinhibitor, Immunostimulator) used in this study.

Supplementary Table 3 Gene list of two immune checkpoint signatures (Inhibitory and Stimulatory) used in this study.

Supplementary Figure 1 Comprehensive analysis of TSPAN4 expression and its prognostic value. **(A)** Violin plot depicting TSPAN4 expression levels in subgroups with different clinical characteristics in CGGA mRNAseq_325 cohort and **(B)** CGGA mRNAseq_693 cohort. **(C)** Kaplan Meier curve showing the overall survival of the high-expression and low-expression groups of TSPAN4 in CGGA mRNAseq_325 cohort and **(D)** CGGA mRNAseq_693 cohort.

## Glossary

ACC: Adrenocortical carcinoma
BLCA: Bladder Urothelial Carcinoma
BRCA: Breast invasive carcinoma
CESC: Cervical squamous cell carcinoma and endocervical adenocarcinoma
CHOL: Cholangiocarcinoma
COAD: Colon adenocarcinoma
COADREAD: Colon adenocarcinoma/Rectum adenocarcinoma
DLBC: Lymphoid Neoplasm Diffuse Large B-cell Lymphoma
ESCA: Esophageal carcinoma
GBM: Glioblastoma multiforme
GBMLGG: Glioma
HNSC: Head and Neck squamous cell carcinoma
KICH: Kidney Chromophobe
KIPAN: Pan-kidney cohort (KICH+KIRC+KIRP)
KIRC: Kidney renal clear cell carcinoma
KIRP: Kidney renal papillary cell carcinoma
LAML: Acute Myeloid Leukemia
LGG: Brain Lower Grade Glioma
LIHC: Liver hepatocellular carcinoma
LUAD: Lung adenocarcinoma
LUSC: Lung squamous cell carcinoma
MESO: Mesothelioma
OV: Ovarian serous cystadenocarcinoma
PAAD: Pancreatic adenocarcinoma
PCPG: Pheochromocytoma and Paraganglioma
PRAD: Prostate adenocarcinoma
READ: Rectum adenocarcinoma
SARC: Sarcoma
STAD: Stomach adenocarcinoma
SKCM: Skin Cutaneous Melanoma
STES: Stomach and Esophageal carcinoma
TGCT: Testicular Germ Cell Tumors
THCA: Thyroid carcinoma
THYM: Thymoma
UCEC: Uterine Corpus Endometrial carcinoma
UCS: Uterine carcinosarcoma
UVM: Uveal Melanoma
OS: Osteosarcoma
ALL: Acute Lymphoblastic Leukemia
WT: High-Risk Wilms Tumor.
