# Supplementary figures and images for: Migrasome regulator TSPAN4 shapes the suppressive tumor immune microenvironment in pan-cancer
Source: Front Immunol. 2024 Dec 11;15:1419420. doi: 10.3389/fimmu.2024.1419420 (PMC11668678; doi:10.3389/fimmu.2024.1419420)

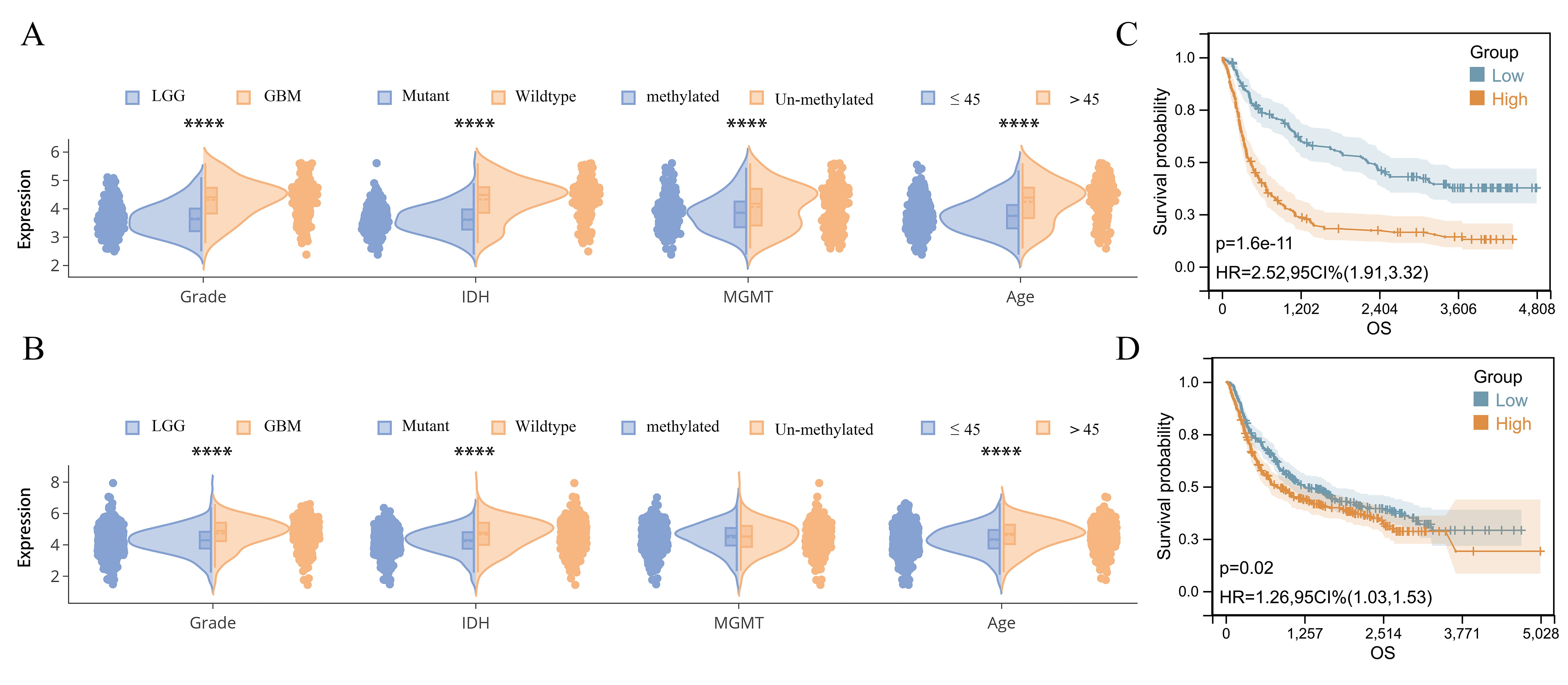

Supplement: Supplementary Figure 1 — Comprehensive analysis of TSPAN4 expression and its prognostic value. (A) Violin plot depicting TSPAN4 expression levels in subgroups with different clinical characteristics in CGGA mRNAseq_325 cohort and (B) CGGA mRNAseq_693 cohort. (C) Kaplan Meier curve showing the overall survival of the high-expression and low-expression groups of TSPAN4 in CGGA mRNAseq_325 cohort and (D) CGGA mRNAseq_693 cohort. [file Image1.tif]
